# Correlation between changes of blood pressure with insulin resistance in type 2 diabetes mellitus with 4 weeks of pioglitazone therapy

**DOI:** 10.4103/0973-3930.41983

**Published:** 2008

**Authors:** L. M. Hettihewa, S. S. Jayasinghe, K. G. Imendra, T. P. Weerarathna

**Affiliations:** Molecular Science and Biomedical Unit, Department of Pharmacology, Faculty of Medicine, University of Ruhuna, Galle, Sri Lanka; 1 Molecular Science and Biomedical Unit, Department of Physiology, Faculty of Medicine, University of Ruhuna, Galle, Sri Lanka; 2 Molecular Science and Biomedical Unit, Department of Medicine, Faculty of Medicine, University of Ruhuna, Galle, Sri Lanka

**Keywords:** Blood pressure, insulin resistance, pioglitazone, type 2 diabetes mellitus

## Abstract

**OBJECTIVE:**

To examine effects of pioglitazone (PIO) on systolic, diastolic, pulse and mean blood pressures (SBP, DBP, PP and MP, respectively) in type 2 diabetes mellitus (T2DM).

**MATERIALS AND METHODS:**

One hundred and six normotensive patients with T2DM with mean fasting blood glucose (FBS; 183 ± 6 mg/dl) were randomly divided into two groups. Test group was treated with 15 mg of PIO in addition to metformin 500 mg three times per day in both groups. SBP, DBP, PP and MP and fasting insulin, FBS and lipid profiles were measured before and after PIO therapy.

**RESULTS:**

There was a significant reduction in SBP (123 ± 2 *vs*. 118 ± 2 mmHg, *P* < 0.05), PP (41 ± 1 *vs*. 37 ± 1 mmHg, *P* < 0.05), and MP (95 ± 1 *vs*. 91 ± 1, *P* < 0.05). Clinical reduction in DBP was observed but not significant (82 ± 2 *vs*. 81 ± 1 mmHg, *P* > 0.05). There was a significant correlation between decline in SBP and DBP with respective baseline values (*r* = 0.76, *P* < 0.001 and *r* = 0.62, *P* < 0.001, respectively). Changes in PP and MP strongly correlated with baseline values (*r* = 0.51, *P* < 0.05 and *r* = 0.56, *P* < 0.05, respectively). There was a parallel reduction of FBS (183 ± 2 *vs*. 121 ± 3, *P* < 0.001) but reduction in IR or lipid profiles was not significant in test group. Changes in BP were not significant in control group ( *P* > 0.05).

**CONCLUSION:**

PIO treatment of T2DM showed early reduction of SBP and MP within first 4 weeks. Results suggest that pharmacodynamic effects of PIO mainly affect the systolic component. We hereby suggest that reduction of BP by PIO is independent from mechanisms of changes in IR and dyslipidaemia in normotensive diabetic patients.

## Introduction

Epidemiological studies have reported that diabetic patients are having an increased prevalence of hypertension, nearly 85% of diabetics are hypertensive and obese by the fifth decade of their lives.[1–3] It is also well documented that diabetic patients are prone to develop microvascular and macrovascular diseases leading to increased mortality.[4–7] Insulin resistance (IR) is frequently associated with hypertension[8–12] and recent studies have shown that thiazolidinedione compounds such as troglitazone, which is an insulin sensitizer, lowers BP in diabetic hypertensives[13] and obese subjects.[14] Because this class of drugs improve insulin sensitivity and glycaemic control, it is not clear whether the BP-lowering effect is due to glycaemic control and improvement in insulin sensitivity or any other mechanism. Recent study by Song *et al.*[15] suggests that troglitazone may improve BP by direct actions on the vasculature. Troglitazone inhibits Ca current in rat's tail artery and aortic vascular smooth muscle cells. Fujishima *et al.*[16] reported that single oral dose of troglitazone increases forearm vasodilatation in healthy volunteers without changing glucose, insulin and BP. Thus, the exact mode of BP-lowering action of PIO is not yet clear.

This study was designed to find out relationship if any, or a common mechanism between improvement of IR and lipid levels with changes in SBP, DBP PP and MBP of type 2 diabetic patients by PIO treatment.

## Materials and Methods

This study was approved by the ethics, research and higher degree committee in the University of Ruhuna prior to the commencement. Patient with T2DM who were on PIO with metformin (test group) or metformin alone (control group) were recruited from hospital medical clinics. Clinical history was obtained from patients including age, sex, medical history, drugs, smoking and alcohol consumption, level of physical exercise, details of DM, coronary heart disease, stroke or peripheral vascular disease. All subjects were non-smokers (never smoked or absence of smoking for at least preceding 2 years) and dietary habits, body weight and physical activity had been stable during preceding 3 months. The following exclusion criteria were used in this study; age outside the range of 20-65 years, hypothyroidism; liver, kidney and heart failure and neoplasia. They were not on any type of antihypertensive therapy. Informed written consent was obtained prior to the commencement of study.

In all participants, BP was measured after 10-min rest period and two readings were recorded at 5-min intervals. BP was measured with a mercury sphygmomanometer in the supine position after 10 min of rest using an appropriate cuff. PP was calculated by obtaining the difference between SBP and DBP and the MBP was calculated by reducing 1/3^rd^ of DBP from SBP in all patients. Body weight and BMI were measured using standard methods. Blood samples (5 ml) were collected after a 12-h overnight fast and serum was separated immediately by refrigerated centrifugation at 4000 rpm. After completion of the above procedure, subjects were placed on 15 mg daily dose of PIO alone in test group and FBS (Diagnostica Merck), fasting insulin (FI; ELISA, Diagnostic Automation), total cholesterol (TC), triglycerides (TG), LDL and HDL (Lab kit, P&T Diagnostics) levels were measured at fortnightly interval. IR of these patients was assessed in each patient by McAuely (McA), HOMA and QUICKI. McA, HOMA and QUICKI were calculated using the following equations.[17–19]

McAuley=exp[2.63−0.28   ln (insulin in mU/1)−0.31   ln (triglycerides in mmol/1)]

HOMA=insulin (μU/ml)×[glucose (mmol/1)/22.5]

$$\text{QUICKI}=\frac{1}{\left(\text{log insulin}+\text{log glycaemia in mg}/\text{dL}\right)}$$

Patients were considered as insulin-resistant when [FI] ≥ 12 mU/l, [McA] ≤ 5.8, [HOMA] ≥ 2.6 and [QUICKI] ≤ 0.33.[17–19]

### Statistical analysis

Power was determined using changes in SBP, DBP, MBP and PP as primary endpoint. For the descriptive statistics after having checked the normality of the variables using the Kolmogorov-Smirnov test, the usual central and dispersion methods were used: average, SD, and 95% CI. All tests were conducted using a probability level of 0.05. The statistical significance of differences between the means were evaluated using the paired Student's *t*-test in the case of normal distribution of data sets, and using the Kolmogorov-Smirnov test when at least in one of the data sets the normal distribution was excluded. Correlation between two variables was studied with the Spearman rank order. All statistical analyses were performed using Microcal Origin 4.1 and Microsoft Excel whenever applicable.

## Results

**Figure 1:**
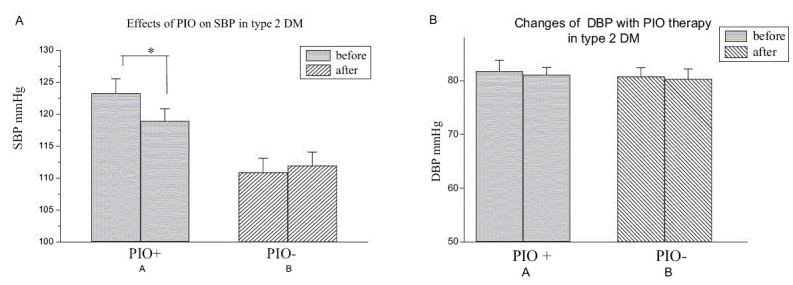
(A) Changes of systolic (SBP) and diastolic (DBP) blood pressure in our patients with type 2 diabetes mellitus with the treatment of 15 mg of pioglitazone for one month. (*n* = 26, *P* = 0.62 for DBP, ^*^P < 0.05 for SBP). (B) Changes observed in diastolic pressure in type 2 diabetes after the treatment with 15 mg of pioglitazone for one month (*n* = 26, *P < 0.05)

**Figure 2:**
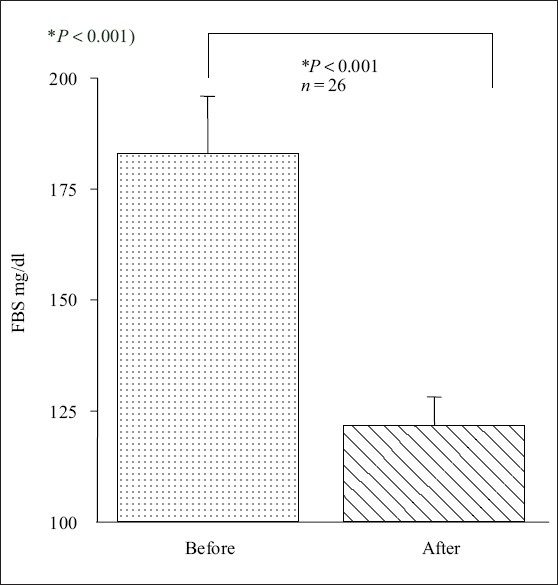
Effect of PIO on FBS in type 2 diabetes (*n* = 26, *P < 0.001)

Baseline demographic data of age, BMI, sex, FI, FBS and values of SBP and DBP of both groups are presented in [Table 1]. Table 2 shows the baseline values of fasting lipid profiles in test group. Table 2 shows that there was no significant difference in lipid profiles and IR measured by three different indirect methods, McA, HOMA and QUICKI after 1 month of PIO. Figure 1A and 1B shows that there was a significant reduction in SBP from 123 ± 2 to 118 ± 1 mmHg (*P* < 0.05). The difference observed in the DBP was not statistically significant (81 ± 2 into 81 ± 1 mmHg, *P* = 0.62). Figure 2 shows that PIO produced a significant decrease in FBS from 183 ± 12 to 121 ± 6 in test group (*P* < 0.001).

**Table 1:** The clinical, anthropometric and metabolic characteristics of test and control groups

Character	Values ± SEM	Values ± SEM
Sample number (*n*)	56	50
Sex (male:female)	11:15	10:16
Age (years)	49 ± 7	48 ± 4
BMI (kg/m^2^)	24.4±0.8	23.7±0.5
FBG (mg/dl)	183.0 ± 12	172 ± 7.1
SBP (mmHg)	123 ± 2	111 ± 1
DBP (mmHg)	81 ± 2	84 ± 3
Fasting insulin level (mU/l)	44.0 ± 5.7	38 ± 5.2

**Table 2:** Changes of lipid profiles and insulin resistance by McAuley, HOMA and QUICKI methods with the pioglitazone treatment in test group

Parameter	Before treatment	After the treatment	Level of significance
Total cholesterol (mg/dl)	250.39 ± 9.9	229.06 ± 5.3	P < 0.005*
Triglycerides (mg/dl)	153.23 ± 9.2	145.8 ± 6.1	P > 0.05
HDL cholesterol (mg/dl)	56.12 ± 2.1	54.06 ± 1.9	P > 0.05
LDL cholesterol (mg/dl)	161.17 ± 9.7	146.03 ± 7.8	P < 0.05*
IR by McAuley	4.44 ± 1.2	4.69 ± 5.3	P > 0.05
IR by HOMA	21.3 ±2.1	14.5 ±1.9	P > 0.05
IR by QUICKI	0.27 ± 0.2	0.28 ± 0.1	P > 0.05

Values are given as mean ± SEM

* Signifcant

There was a significant reduction of PP from 41 ± 1 (before treatment) to 37 ± 1 mmHg in all patients (Figure 1B; *P* < 0.05). Further, the reduction of the MP from 95 ± 1 to 91 ± 1 mmHg was statistically significant (*P* < 0.05).

These results indicate that the changes in BP were mainly due to the decrease in the SBP resulting parallel reduction of PP. Further, we found that there was a significant correlation between decline in SBP and DBP with respective baseline values after the treatment with PIO (*r* = 0.76, *P* < 0.001 and *r* = 0.62, *P* < 0.001, respectively; data not shown). In addition, our data show the strong correlation of decline in PP and MP with the baseline values (*r* = 0.51, *P* < 0.05 and *r* = 0.56, *P* < 0.05, respectively; data not shown).

**Figure 3:**
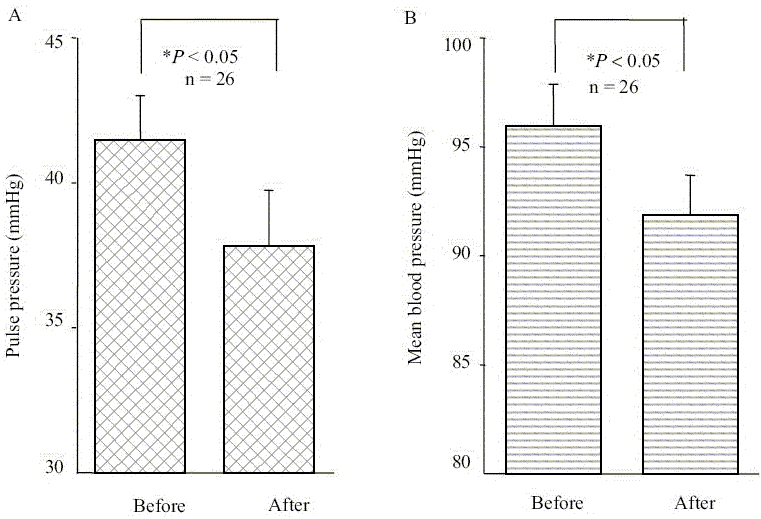
(A) Effects of PIO on pulse pressure in type 2 diabetes (B) Effects of PIO on mean pressure changes in type 2 diabetes

Our results are in agreement with previous findings with troglitazone by Sung *et al.*[20] and rosiglitazone by Raj *et al.*[21] To understand the possible underlying mechanism of clinically effective reduction in SBP, PP and MP in our study group, we investigated whether the changes observed in SBP, PP and MP were correlated to the reduction of IR in these patients. As described in section ‘Materials and Methods’, we investigated for any correlation between reduction of BP with the changes in total cholesterol, LDL, HDL, triglycerides or with indirect methods of detecting IR (MCA,[17] HOMA[18] and QUICKI[19]). Figure 3A and B shows the correlation between McA and either with changes of SBP or changes of DBP is not statistically significant. Our result further shows that there is no significant correlation of BP changes with any other parameter mentioned earlier (data not shown).

## Discussion

**Figure 4:**
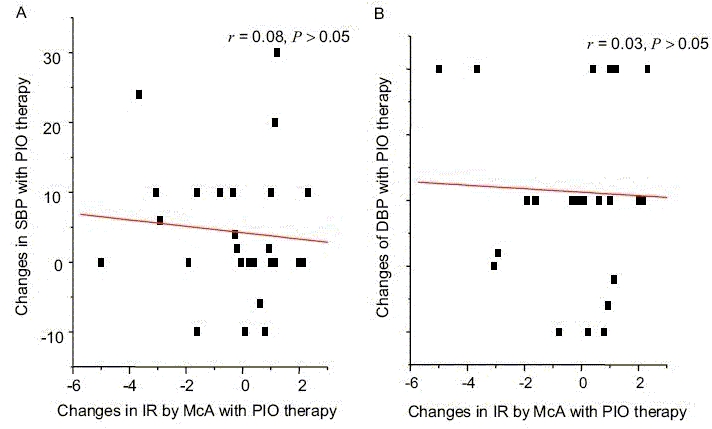
(A) Correlation between changes in insulin resistance (MCA) with changes in SBP. (B) Correlation between changes in insulin resistance (MCA) with changes in DBP

Many previous studies have reported that essential hypertension is accompanied by IR, dyslipidaemia, diabetes and other cardiovascular risk factors.[22–25] However, it is not well established whether certain groups of hypertensive patients among diabetic population are more insulin-resistant than others or whether treatment with an insulin sensitizer might effectively lowers the blood pressure. Raj *et al.* demonstrated subjects with salt sensitive essential hypertension are more than low rennin hypertensive group.[21] The current study was designed to determine the effects of PIO on SBP, DBP, PP and MP as well as to find out the correlation between cardiovascular risk factors (risk lipid levels) with BP changes. Our study confirms that PIO treatment in type 2 diabetic patients had early effects on reduction of FBG. Our patients are not hypertensive at the beginning of the treatment. There was a statistically significant reduction in SBP, PP and MBP within first 4 weeks of therapy in normotensive type 2 diabetic patients. Though the reduction observed in DBP is not statistically significant, difference was clinically remarkable. According to Figure 1A and B, our data show that changes in PP and MP are parallel with their baseline values. This further suggests that there is a reduction in systemic blood pressure with the treatment of PIO. Although we observed a significant reduction of FBG levels in our study group [Table 2], changes observed in lipid profiles or IR within first 4 weeks of PIO therapy are not statistically significant. Further, we could not correlate these changes in BP with any of the lipid parameter or with the IR [Figure 4A and B]. Considering all these facts, we suggest that possible pharmacological action of PIO on reduction of BP, mainly affecting SBP, does not involve the mechanisms of dyslipidaemia and IR. We think that there is a possibility of a common receptor or a different mode of action for the observed changes in BP and the FBS. Our results are compatible with Reaven and Banting,[23] showing that hypotensive effects of troglitazone is independent of the mechanism of dyslipidaemia (Ca^++^-dependant smooth muscle relaxation) and IR. Although BP-lowering effects have been reported,[18] the underlying haemodynamic mechanism has not been well delineated. Researchers have identified a molecule that binds to a receptor in the brain known to regulate blood pressure and stimulates similar receptors in the pancreas that regulate release of insulin.^[28]^ The discovery may lead to simultaneous treatment options for high blood pressure and diabetes. The newly discovered molecule acts as a neurotransmitter and thus conveys messages throughout different regions of the brain. There was a significant reduction in FBS and SBP, PP and MP in our study group within 4 weeks of therapy and no significant reduction in FI or lipid profiles. This result is supported by above researchers,[28] which shows there is an association of regulating insulin release and control of BP in diabetic patients. Fujishima *et al.*[15] have reported that single dose of troglitazone increased forearm vasodilatation in healthy volunteers without changes in glucose, insulin and BP, which excludes the mechanism of maintenance of glucose and insulin levels. Size of the study sample in our work was small to predict it to the population level.

Lowering of blood pressure in diabetic population is particularly important for prevention of progression of DM and complications. The Joint National Commission VI recommends treating high-normal BP in the diabetic population.^[27]^ We suggest mechanism of BP-lowering effect of PIO may be independent from the mechanism-causing IR as well as dyslipidaemia in normotensive diabetic patients. Work has to be expanded to investigate the observed effect of PIO on reducing BP also in type 2 diabetic patients who are complicated with hypertension too.
